# Defoliation in Perennial Plants: Predictable and Surprising Results in *Senna* spp.

**DOI:** 10.3390/plants12030587

**Published:** 2023-01-29

**Authors:** Suzanne Koptur, Andrea Salas Primoli, María Cleopatra Pimienta

**Affiliations:** Department of Biological Sciences, International Center for Tropical Botany, Institute of the Environment, Florida International University, Miami, FL 33199, USA

**Keywords:** compensation, defense, defoliation, extrafloral, Fabaceae, foliar, induction, legume, nectaries

## Abstract

When some plants are defoliated, they may suffer by reaching a smaller final size than if they had not been damaged. Other plants may compensate for damage, ending up the same size as if they had not been damaged. Still, others may overcompensate, ending up larger after defoliation than if they had been spared from damage. We investigated the response of *Senna* species (Fabaceae) to defoliation, comparing two native and several ornamental congeners, all of which grow locally in southern Florida. Many *Senna* spp. bear foliar nectaries as nutritional resources for beneficial insects that may, in exchange, protect them from herbivores. We grew five species from seed and subjected them to three levels of defoliation for a period of several months to measure effects of leaf area removal on plant height, number of leaves, and number of extrafloral nectaries. Only three of five species displayed shorter plant heights with greater levels of damage. Two species produced fewer new leaves with moderate to severe defoliation. In only one species, the number of extrafloral nectaries decreased with defoliation, suggesting that while extrafloral nectar production may be an inducible defense in some species, producing more nectaries in response to damage does not occur in these *Senna* species.

## 1. Introduction

Plants are autotrophs, using the sun’s energy and carbon dioxide to produce sugars and energy for their growth and structures. They are food for herbivores, a conduit for that energy from the sun to the next trophic level. Herbivores, primary consumers in most food webs, come in all shapes and sizes, and eat plants in various ways [1]. The most visible loss of tissues happens when parts are consumed, such as leaves, flowers, and fruits removed by animals. 

What happens when plants are eaten? If entirely consumed, a plant will die, and if its biomass is greatly reduced but not enough energy was stored, the damage may also inflict mortality. If tissues are lost to herbivores, the amount of photosynthate produced is reduced, and plants may show reduced growth [2] or reproduce less [3,4]. Depending on the life history and habitat of the plant, the effects may be expressed the same, or the following, year [5,6,7]. Some damaged plants may regrow to the size of uneaten plants; compensation for herbivory [8,9] is facilitated by greater root/shoot ratios [10], storage organs [11,12], or by upregulation of the plant’s physiological responses and growth [13,14]. Additionally, other plants may regrow even larger or reproduce more than uneaten plants, a phenomenon called over-compensation [15]. 

*Senna* species are perennial plants in the Fabaceae family, with life forms from herbs, shrubs, to small trees. They serve as host plants for sulphur butterflies (Pieridae), whose caterpillars may at times defoliate them severely [16,17]. In south Florida, two *Senna* species are native: *Senna mexicana* occurs in pine rocklands, and *S. ligustrina* occurs in wet prairie habitats. Both are used in native plant landscaping. With their bright green foliage and yellow flowers, other exotic species are used in landscaping as well, including the candlestick plant (*S. alata*), the desert senna (*S. polyphylla*), and golden senna (*S. surattensis*). All species are used as host plants by butterflies in southern Florida [18].

Many *Senna* species (including those mentioned above) bear foliar nectaries [19] on their compound leaves [20], which serve to attract ants and other beneficial insects [21,22,23,24], though those insects may not always provide defense [25]. Studies on *S. mexicana* var. *chapmannii* have shown that nectar production is greater at night (as in many other species, e.g., [26]), older plants produce more nectar than younger plants [27], and nectaries are not present on seedlings. Nectar production is inducible, elevated in response to leaf damage [27], as has been found in other plants [28,29,30,31,32,33,34]. 

Extrafloral nectar provides effective biotic defense against herbivory in *S. mexicana*: when ants are excluded from plants, there are more caterpillars eating the leaves and flowers [17]. The presence of ants increases *Senna* plant reproductive fitness, but only in sunny habitats [35], as has been found in other plants bearing extrafloral nectaries [36]. The plants with ants grew larger, had bigger floral displays, attracted more pollinators, and set more fruit [37]. Studies with other plants have found that some respond to defoliation by producing more nectaries on leaves produced after damage [29,38,39,40,41].

We sought to compare the short-term responses of congeneric species of *Senna* (Fabaceae) to artificial defoliation. By subjecting individual seedlings to continuing levels of leaf loss over their development, we could see if and how defoliation affected their growth, production of new leaves, and the number of extrafloral nectaries on their foliage. Our questions were:

(1) Will plants experiencing greater levels of defoliation grow less than those with less damage?
(2) Will plants experiencing greater levels of defoliation produce fewer new leaves?
(3) Will plants experiencing greater levels of defoliation produce more foliar nectaries?

The null hypotheses are that the amount of leaf damage will have no effect on *Senna* plant height, number of new leaves produced, or number of extrafloral nectaries. Alternatively, these variables may increase or decrease in comparison to the non-defoliated controls. 

Our expectation was that *Senna* species might differ in their responses, related perhaps to their relative sizes. The same amount of damage on a smaller plant may have a greater impact than that amount on a larger plant. We predicted that larger *Senna* plants might compensate for defoliation and grow to sizes similar to undamaged plants. We also predicted they might produce more foliar nectaries on more heavily defoliated plants.

## 2. Results

**Figure 1:**
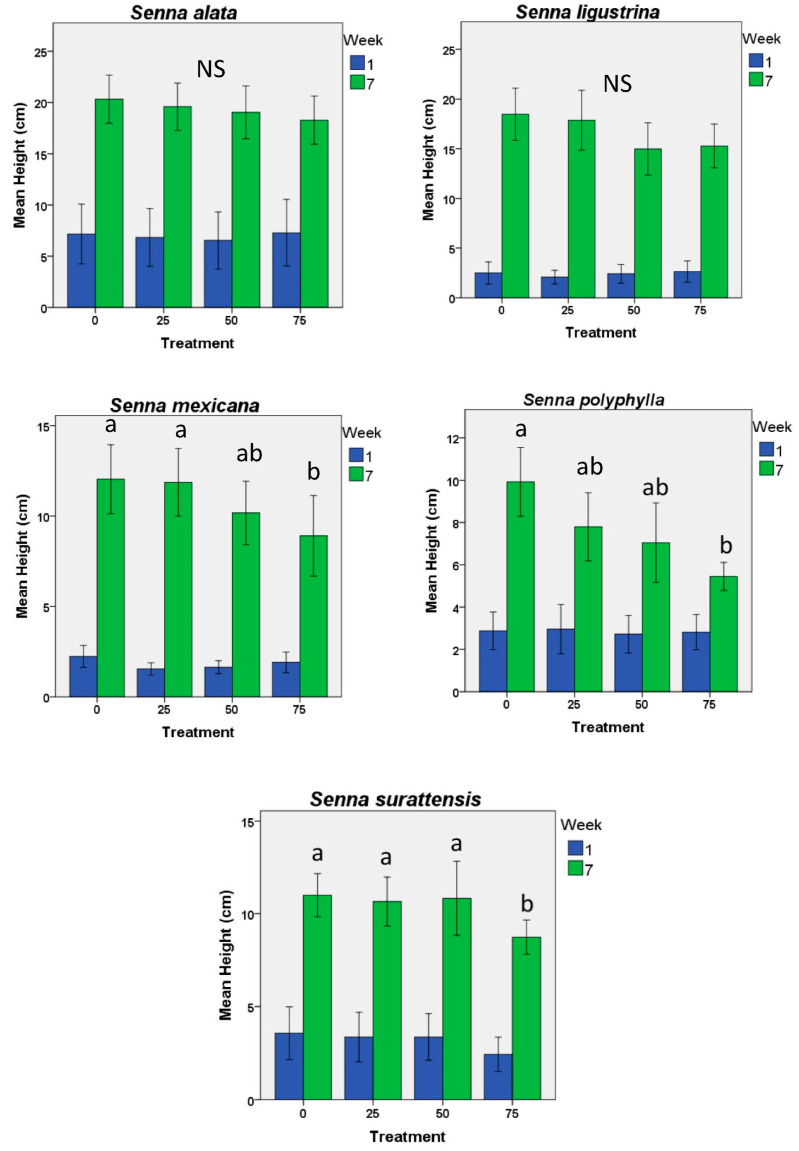
Heights—mean (+2 S.E.) beginning and final heights of five species of *Senna* subject to different levels of artificial defoliation over the first two months of life (n = 22 plants each level and control). In no case were the initial heights different among the treatment groups. Small letters by bars indicate significant differences (*p* < 0.05); bars with the same letters do not differ significantly from each other. NS indicates no significant differences found.

For three of the five species (*S. mexicana, polyphylla*, and *surattensis*), plants were shorter with higher levels of defoliation (Figure 1). The other two species (*S. ligustrina* and *S. alata*) showed no effects of defoliation on their final heights.

**Figure 2:**
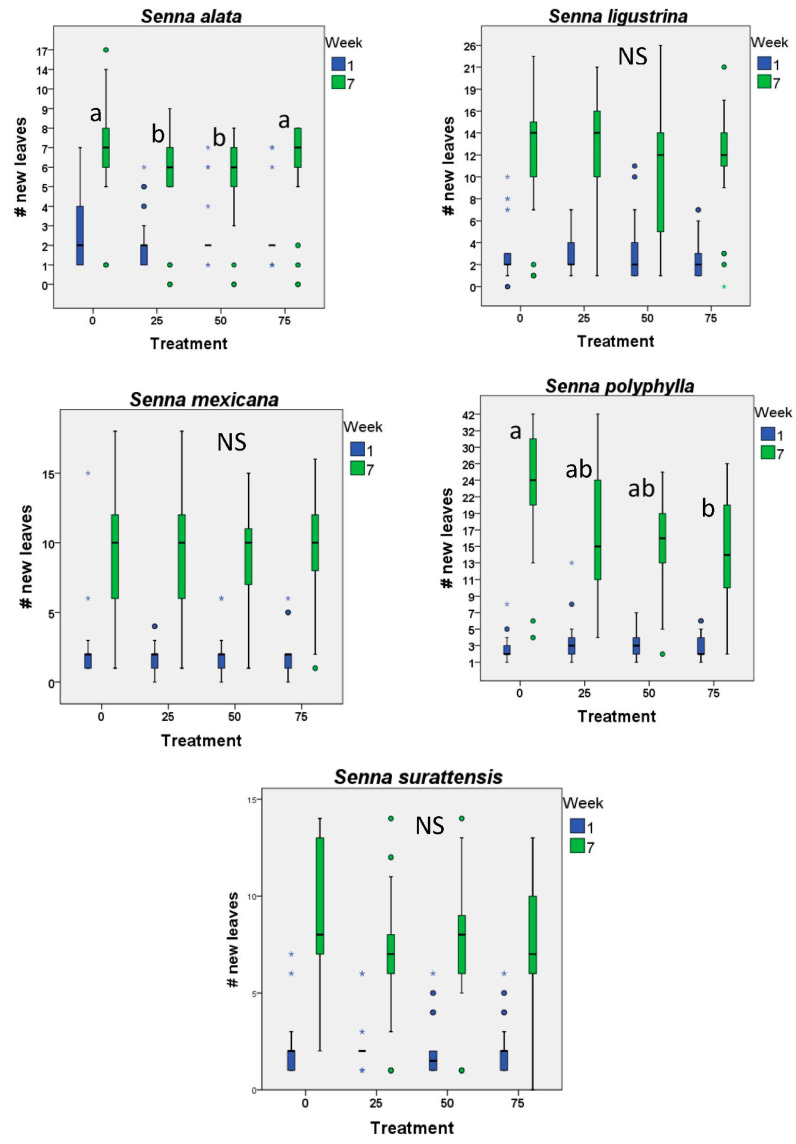
New leaves—bar and whisker plots for initial number of leaves and final number of new leaves produced on five species of *Senna* subject to different levels of artificial defoliation over the first two months of life (n = 22 plants each level and control). In no case were the initial number of leaves different among the treatment groups. Small letters by bars indicate significant differences (*p* < 0.05); bars with the same letters do not differ significantly from each other. NS indicates no significant differences found.

Only one species (*S. polyphylla*) produced fewer new leaves with higher levels of defoliation. Interestingly, *S. alata* produced fewer new leaves at intermediate levels of defoliation. All other species seemed to compensate for lost leaf area, producing the same number of new leaves as undamaged controls (Figure 2).

**Figure 3:**
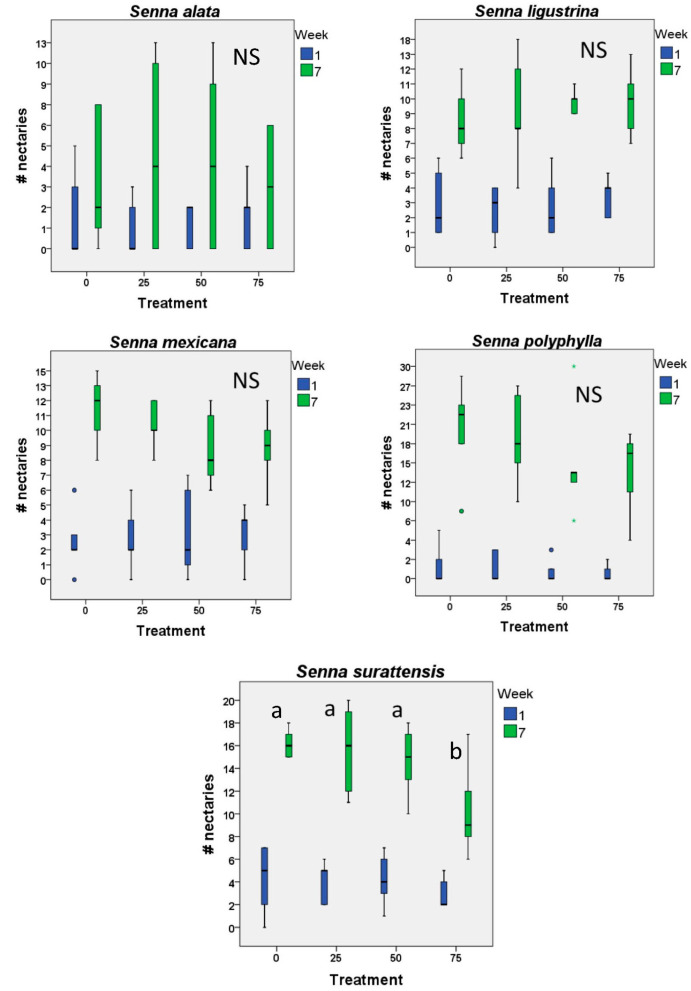
Extrafloral nectaries—bar and whisker plots for initial and final number of extrafloral nectaries on five species of *Senna* subject to different levels of artificial defoliation over the first two months of life (n = 12 plants each level and control). In no case were the initial number of nectaries different among the treatment groups. Small letters by bars indicate significant differences (*p* < 0.05); bars with the same letters do not differ significantly from each other. NS indicates no significant differences found.

We have data for the number of nectaries from only one year, and just one species (*S. surattensis*) produced significantly fewer nectaries with higher levels of defoliation (Figure 3). Perhaps with a larger sample size, we might have seen a significant difference in other species that showed a similar trend (*S. mexicana* and *S. polyphylla*).

## 3. Discussion

The three *Senna* species that showed decreased height with severe defoliation apparently compensated for mild and moderate defoliation. Our results demonstrate that *Senna* spp. are more resilient to defoliation than, for example, *Mimosa pigra* [42], which could only compensate with mild defoliation. 

The species of *Senna* that produced fewer new leaves in response to higher levels of defoliation apparently had less ability to compensate for leaf area lost, perhaps due to having less storage in their non-photosynthetic parts. To better understand this possibility, the biomass root/shoot ratio of the species could be compared. 

The only species that produced fewer extrafloral nectaries was *S. surattensis*, the one that bears multiple nectaries per leaf; all other species we studied have only one. Multiple nectaries may be a more labile trait and are a characteristic of other species that have been found to produce more nectaries with defoliation, such as *Mallotus japonicus* (Euphorbiaceae) [41], *Sapium sebiferum* (Euphorbiaceae) [29], *Prunus avium* (Rosaceae) [40], and *Vicia faba* (Fabaceae) [38,43]. However, the response of *S. surattensis* was to produce fewer, not more, foliar nectaries with higher levels of defoliation. Rather than the formation of nectaries being an inducible defense in *Senna*, it may be a feature that can be cut back as defoliation limits energy resources. It has been shown that greater extrafloral nectar production is induced with defoliation in *S. mexicana* [27], but in the present study, nectar quantity was not measured. In a future study, it will be worth investigating if this is a general response among *Senna* species. 

A similar field experiment with *Qualea multiflora* in the Brazilian *cerrado* showed that higher levels of defoliation led to greater nectar production and greater ant attendance [44]. Plants with more and better nectar receive greater levels of defense, as demonstrated in another field experiment involving a single species of ant and its predation on termites on five different plant species [45], and greater amounts of extrafloral nectar have a direct positive effect on ant colony fitness [46].

Our results must be interpreted with some caveats in mind. With less than two months of growth, we cannot be certain that greater differences among treatments might not have emerged over time. Many other studies have followed plants over an entire growing season, or more. Furthermore, the timing of defoliation can have substantially different effects on subsequent growth and reproduction as found in *Sesbania* (Fabaceae) [4], as well as their survival, as seen in *Prosopis* (Fabaceae) [47], though in some plants it does not make a difference [44]. 

This study was performed on potted plants in a greenhouse, not in nature. As Agrawal [48] observed: “Resource limitation is only half of understanding trade-offs…the rest is community ecology”. Subtle differences in plant stature and leaf number may be more meaningful when a plant grows with neighbors of the same and other species [49], so that responses among individuals may be significant for those individuals even if the average response seen in isolated plants may not be. 

Our defoliations were artificial, performed with scissors, not real herbivores; in some species, this has been shown to make a difference. Real herbivores had a greater effect on eucalyptus seedlings than did artificial defoliation [50]. Similar comparisons with *Mimosa pigra* (Fabaceae) showed no difference [42]. 

Some studies have found greater sensitivity to defoliation in non-native vs. native species [51], though we did not see this difference in the congeneric species in this study. While sulphur butterflies oviposit on all the *Senna* spp. we studied, there is no apparent preference for either native or non-native species (Koptur and Salas, unpublished data), despite the general findings that non-native species suffer greater defoliation than natives in a given habitat [52]. 

Our challenge now is to learn how different levels of defoliation affect the growth, competitive ability, and reproduction of these plants in nature. While the sulphur butterflies, *Phoebis* spp. (Pieridae), normally remove leaf material from *Senna* plants to levels of less than 25%, the sleepy orange butterfly (*Abaeis nicippe*, Pieridae) oviposit prolifically and the caterpillars may defoliate plants to levels in excess of 80% [16]. Our greenhouse experiments and previous field experiments [17,18,19,35,37] have controlled for surroundings and many of the interactions naturally occurring plants experience. We hope these will enable us to better interpret the responses observed in nature. 

## 4. Materials and Methods

### 4.1. Study Species

**Figure 4:**
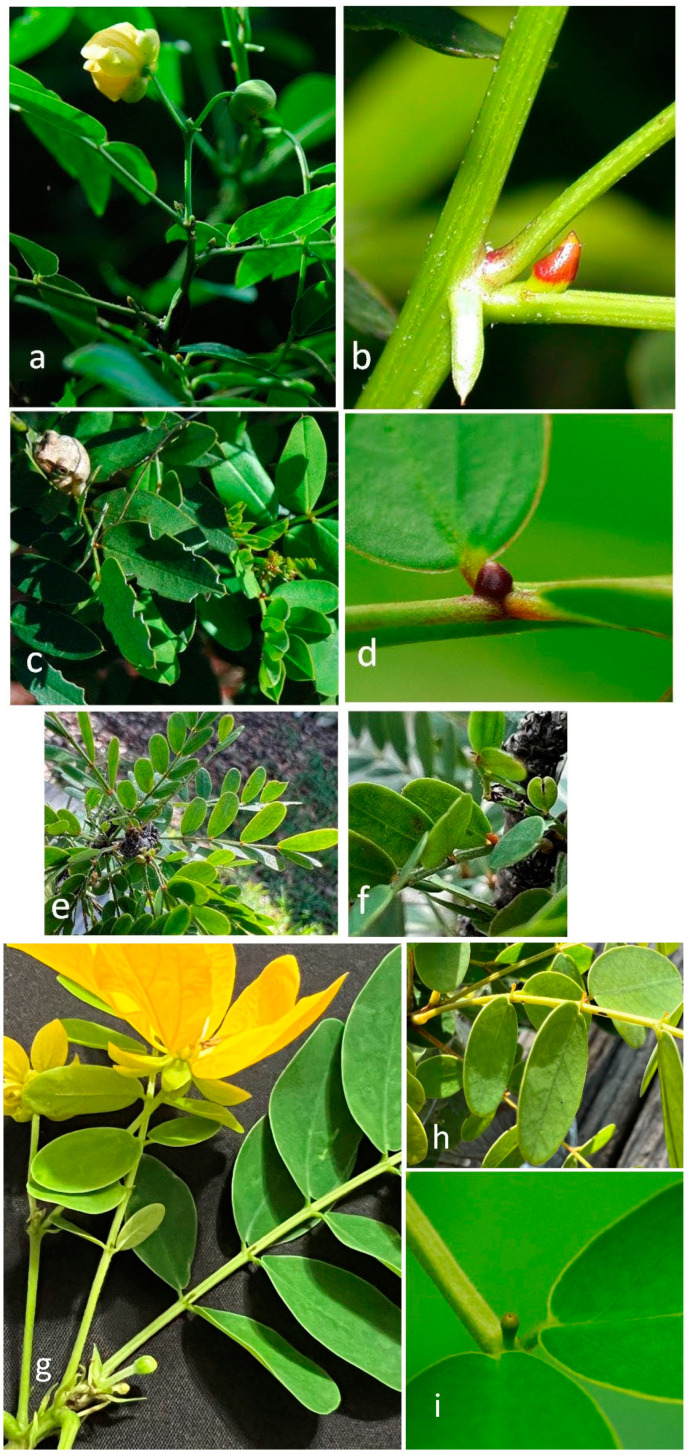
*Senna* species and nectaries—(**a**) *Senna ligustrina* habit, (**b**) closeup of *S. ligustrina* nectary at base of leaf; (**c**) *S. mexicana* var. *chapmanii* habit, (**d**) closeup of *S. mexicana* nectary between lowest pair of leaflets; (**e**) *S. polyphylla* habit, (**f**) closeup of *S. polyphylla* nectary between lowest pair of leaflets. (**g**) *S. surattensis* habit, leaf with two foliar nectaries between basal leaflet pairs; (**h**) *S. surattensis* leaf with three nectaries, exuding nectar; (**i**) close-up of single nectary of *S. surattensis* between a pair of leaflets.

We investigated five species of *Senna* (Figure 4), two native to south Florida and three exotic species. The two native species are similar in general appearance of leaves and flowers, but the leaflets and foliar nectaries of *Senna ligustrina* (L.) H.S. (Irwin and Barnaby) are pointed, whereas those of *S. mexicana* var. *chapmannii* (Isely) H.S. (Irwin and Barnaby) are rounded. Naturally occurring in wetlands, *S. ligustrina* grows as an upright, multi-stemmed shrub reaching 2 m in height, whereas *S. mexicana* plants in pine rocklands are lower-growing (normally 1 m or less in height) and tend to sprawl laterally.

The three exotic species are quite different in appearance and habit. A big, robust shrub with large sprawling branches up to 2 or more meters tall, *Senna alata* (L.) Roxb. has very large leaves with glandular stipules [53,54], and perhaps glandular areas between leaflets as well, nectaries termed non-individualized nectaries by Marazzi et al. [55]. A more delicate shrub with small leaves and leaflets, *S. polyphylla* (Jacq.) (Irwin and Barnaby) has nectaries between the lowest pair of leaflets; they can grow up to be small trees up to 3 m in height. Commonly planted as a street tree in parking lots, on roadsides, and medians, *S. surattensis* (Burm. F.) (H.S. Irwin and Barnaby) has medium-sized leaves with rounded leaflets and nectaries between the lower pairs of leaflets. 

### 4.2. Experimental Design

The experiment was conducted in spring of 2017 and spring of 2019 in the greenhouse facility of the Biology Department of Florida International University. Starting with bulk samples of seeds collected from at least four individuals of plants of five *Senna* species growing in south Florida, we scarified the seeds and soaked them overnight. Seeds were planted in standard greenhouse soil and germinated within two weeks of planting, growing to transplant size within a month. 

Seedlings were potted individually in 4” pots and arranged in blocks in which all five species were each represented by four plants. One individual of each species was assigned to one of the following experimental treatments: no defoliation control, 25% defoliation, 50%, and 75% defoliation. These levels encompass the range of damage individual leaves and plants may experience in nature. On individual plants, each leaf was defoliated to the designated level, with new leaves defoliated each week. For this artificial defoliation, we used sharp scissors to simulate damage from externally feeding caterpillars.

After seven weeks, we measured plant height, the number of leaves, and number of nectaries (only in 2017) present on each individual plant. Data were combined for the two years to provide robust sample sizes. Twelve blocks were utilized in 2017, and ten in 2019, giving a total sample size of 22 plants in each treatment for each species. Plant height, a continuous variable, was compared among treatments with analysis of variance. The number of new leaves produced and the number of nectaries, count data, were compared with the non-parametric Kruskal-Wallis test.

## Data Availability

Data may be freely accessed from the FIU dataverse: https://doi.org/10.34703/gzx1-9v95/VNZZJZ (accessed on 1 December 2022).
